# Genetic diversity and population structure analysis to construct a core collection from a large *Capsicum* germplasm

**DOI:** 10.1186/s12863-016-0452-8

**Published:** 2016-11-14

**Authors:** Hea-Young Lee, Na-Young Ro, Hee-Jin Jeong, Jin-Kyung Kwon, Jinkwan Jo, Yeaseong Ha, Ayoung Jung, Ji-Woong Han, Jelli Venkatesh, Byoung-Cheorl Kang

**Affiliations:** 1 Department of Plant Science and Vegetable Breeding Research Center, Seoul National University, Seoul, 151-921 Korea; 2 National Academy of Agricultural Science, Rural Development Administration, Jeonju, 560-500 Korea

**Keywords:** *Capsicum* spp., Core collection, Genetic diversity, Germplasm, Population structure

## Abstract

**Background:**

Conservation of genetic diversity is an essential prerequisite for developing new cultivars with desirable agronomic traits. Although a large number of germplasm collections have been established worldwide, many of them face major difficulties due to large size and a lack of adequate information about population structure and genetic diversity. Core collection with a minimum number of accessions and maximum genetic diversity of pepper species and its wild relatives will facilitate easy access to genetic material as well as the use of hidden genetic diversity in *Capsicum*.

**Results:**

To explore genetic diversity and population structure, we investigated patterns of molecular diversity using a transcriptome-based 48 single nucleotide polymorphisms (SNPs) in a large germplasm collection comprising 3,821 accessions. Among the 11 species examined, *Capsicum annuum* showed the highest genetic diversity (H_E_ = 0.44, I = 0.69), whereas the wild species *C. galapagoense* showed the lowest genetic diversity (H_E_ = 0.06, I = 0.07). The *Capsicum* germplasm collection was divided into 10 clusters (cluster 1 to 10) based on population structure analysis, and five groups (group A to E) based on phylogenetic analysis. *Capsicum* accessions from the five distinct groups in an unrooted phylogenetic tree showed taxonomic distinctness and reflected their geographic origins. Most of the accessions from European countries are distributed in the A and B groups, whereas the accessions from Asian countries are mainly distributed in C and D groups. Five different sampling strategies with diverse genetic clustering methods were used to select the optimal method for constructing the core collection. Using a number of allelic variations based on 48 SNP markers and 32 different phenotypic/morphological traits, a core collection ‘CC240’ with a total of 240 accessions (5.2 %) was selected from within the entire *Capsicum* germplasm. Compared to the other core collections, CC240 displayed higher genetic diversity (I = 0.95) and genetic evenness (J’ = 0.80), and represented a wider range of phenotypic variation (MD = 9.45 %, CR = 98.40 %).

**Conclusions:**

A total of 240 accessions were selected from 3,821 *Capsicum* accessions based on transcriptome-based 48 SNP markers with genome-wide distribution and 32 traits using a systematic approach. This core collection will be a primary resource for pepper breeders and researchers for further genetic association and functional analyses.

**Electronic supplementary material:**

The online version of this article (doi:10.1186/s12863-016-0452-8) contains supplementary material, which is available to authorized users.

## Background

Pepper (*Capsicum* spp.) is one of the major vegetable and spice crops grown worldwide, and is rich in bioactive compounds, such as capsaicinoids and carotenoids, which contribute to the improvement of human health [1, 2]. Because of its economic and nutritional importance, breeders have improved agronomic traits of pepper, such as pungency, fruit shape, abiotic stress tolerance, and disease resistance. Meanwhile, genetic diversity of breeding lines has become smaller and some useful genes in the landraces are lost due to the breeding activities [3, 4]. Therefore, conservation and sustainable utilization of genetic resources are keys to continuous improvement of peppers [5].

During the last several decades, there has been remarkable progress in germplasm collection and conservation of various plants. Although a large number of germplasms have been collected, their management has become more and more complicated due to their huge sizes. Furthermore, little is known about the genetic diversity and structure of such collections at the interspecific and intraspecific levels [6]. To make efficient use of large germplasm collections, the concept of core collections has been proposed. A core collection is a subset of a germplasm collection of a species that represents the genetic diversity of the entire collection [7]. A good core collection is one that has no redundant accessions, is small enough to be easily managed, and represents the total genetic diversity [8].

Various types of data including passport data, geographic origin [9, 10], agronomic traits [11–13], and molecular markers [14] can be used for selecting a core set. Although the major reason for establishing a core set is to reduce the number of representative accessions up to 10 % while maintaining the diversity of the entire collection, there are a number of possible methods for selection of a core set depending on the research goals. In the early 2000s, most researchers performed random sampling using various assignment methods [9, 11]. Later, the M (maximization) strategy was proposed as a more effective method to select a core set representing the maximum genetic diversity without redundancy [12, 15].

Several research institutions have collected and conserved thousands of *Capsicum* accessions, ranging from 1,000 in the Centre for Genetic Resources (CGN), the Netherlands [16] to almost 8,000 in the Asian Vegetable Research and Development Center (AVRDC), Taiwan [17]. Researchers and institutions have attempted to construct core collections of *Capscicum* spp. for various purposes. Fan et al. [13], Nicolai et al. [14], and Zewdie et al. [12] established core collections to reveal phenotypic and genetic variation. Thies and Fery [9], and Quenouille et al. [10] constructed a core collection for disease resistance against northern root-knot nematode and *Potato virus Y* (PVY), respectively. Hanson et al. [11] developed a core collection to analyze antioxidant activities. However, most studies involved a relatively small number of accessions, using fewer than 1,000 accessions with limited numbers of morphological traits and molecular markers [11, 12, 14]. The limited number of morphological traits and markers allow us to survey only a small portion of the genetic diversity of the entire germplasm, and the resulting data cannot be used for genome-wide variation studies.

In this study, we performed population structure analysis in a large *Capsicum* germplasm collection consisting of 3,821 accessions by applying 48 genome-wide SNPs, and selected a core set using the SNP data together with data for 32 morphological traits. This allowed us to 1) examine the level of genetic diversity and the population structure within the worldwide *Capsicum* germplasm collection; 2) optimize selection methods by comparing different core sets, which were selected using a stepwise selection strategy based on various combinations of data and clustering methods; and 3) ultimately construct a *Capsicum* core collection that represents the entire germplasm collection without redundancy. Finally, we validated the core collection by evaluating the diversity of a range of traits and genotyping additional molecular markers. This core collection will be a valuable data set for both pepper breeding and genome-wide association studies.

## Methods

### Plant materials

A total of 4,652 *Capsicum* accessions used in this study originated from 97 countries and included 11 species: *C. annuum*, *C. baccatum*, *C. cardenasii*, *C. chacoense*, *C. chinense*, *C. eximium*, *C. frutescens*, *C. galapagoense*, *C. praetermissum*, *C. pubescens*, and *C. tovarii*. The geographic origin and passport data of the germplasm accessions were obtained from the Rural Development Administration (RDA, Jeonju, Korea) and Seoul National University (SNU, Seoul, Korea). Among the germplasm accessions, 3,599 were obtained from the RDA, and 1,053 were obtained from SNU. Most of the accessions were *C. annuum*, accounting for 4,163 accessions. Four other domesticated species, *C. baccatum*, *C. chinense*, *C. frutescens*, and *C. pubescens* accounted for 163, 122, 152, and 11 accessions, respectively. Among the wild *Capsicum* species, *C. cardenasii*, *C. chacoense, C. eximium*, *C. galapagoense*, *C. praetermissum* and *C. tovarii* accounted for 1, 28, 4, 2, 5, and 1 accessions, respectively.

### DNA extraction and SNP genotyping

Two young leaves from each accession were used for DNA extraction. DNA was extracted using the cetyl trimethylammonium bromide (CTAB) method as described previously [18]. The concentration and purity of DNA samples were determined with a NanoDrop 1000 spectrophotometer (NanoDrop Technologies, Wilmington, DE, USA). DNA samples showing absorbance ratios above 1.8 at 260/280 nm were used for marker analysis.

A set of 48 SNP markers evenly distributed in 12 pepper chromosomes were used in this study [19] (Additional file 1: Table S1). In a preliminary study a total of 282 accessions were randomly selected from entire germplasm collection for genetic diversity study with 412 SNP markers developed by Kang et al. [19]. Based on this analysis, highly polymorphic SNP markers (PIC > 0.45) were selected. Genotyping was performed using the BioMark™ HD system (Fluidigm, San Francisco, CA, USA), EP1™ system (Fluidigm, San Francisco, CA, USA), and 48 × 48 Dynamic Array IFCs (Fluidigm, San Francisco, CA, USA) according to the manufacturer’s protocol [20]. Specific target amplification (STA) was performed prior to SNP genotyping analysis. PCR was performed in a 5-μL reaction containing 60 ng of the DNA sample according to the manufacturer’s protocol. Thermal cycling conditions were 15 min at 95 °C, followed by 14 cycles of a 2-step amplification profile of 15 s at 95 °C and 2 min at 60 °C. For genotyping, SNPtype assays were performed using STA products following manufacturer’s protocol. Thermal cycling was carried out at 95 °C for 15 s, 64 °C for 45 s and 72 °C for 15 s with a touchdown of −1 °C per cycle from 64 to 61 °C, followed by 34 cycles of 95 °C for 15 s, 60 °C for 45 s and 72 °C for 15 s. For the species verification and/or identification of pepper accessions with missing species information, SNP markers C2_At5g04590, C2_At1g50020, and C2_At2g19560 were used based on high resolution melting (HRM) analysis [21]. Genotyping analysis was performed using a Rotor Gene 6000 (Qiagen, Valencia, CA, USA).

### Population structure analysis

To analyze the population structure of the entire germplasm collection used in this study, we used a model based genetic clustering algorithm [22] as implemented in the STRUCTURE program ver. 2.3.4 [23]. The number of sub-populations (ΔK) was determined using the *ad-hoc* statistical method, based on the rate change in the log probability of data between successive K values [24]. Fifty independent runs for K values ranging from 1 to 20 were performed with a burn-in length of 50,000 followed by 1,000,000 iterations.

### Phylogenetic and principal coordinate analyses

Phylogenetic trees were produced using genotyping data with 48 SNP markers using both the unweighted neighbor-joining method and the hierarchical clustering method based on the dissimilarity matrix calculated with Manhattan index, as implemented in the DARwin software (version 6.0.9). Principal coordinate analyses were also performed with DARwin 6.0.9 [25].

### Statistical analysis of genetic diversity

Different indices were used for analysis and comparison of diversity among the *Capsicum* collections. These include levels of observed heterozygosity (H_O_), expected heterozygosity (H_E_), polymorphic information content (PIC), genetic differentiation (F_ST_), Shannon’s information index of diversity (I), and genetic evenness (J’). Indices Ho, H_E_, PIC, and F_ST_ were calculated using Power Marker 3.25 [26]. For analysis of genetic diversity of core collections, I and J’ were calculated following Hennink and Zeven [27] and Pielou [28], respectively. Analysis of molecular variance (AMOVA) was conducted to detect the genetic variance within and among population using GenAlEx ver 6.502 [29].

### Establishment of the core collection

To establish a core collection, five different methods were used. Specifically, core sets were selected based on 1) genotype analysis of the entire collection, 2) genotype analysis of each cluster after grouping based on genotype dissimilarity, 3) phenotype analysis of the entire collection, 4) a combination of genotype and phenotype analysis of entire collection, and 5) a combination of phenotype and genotype analysis of each cluster after grouping based on genotype dissimilarity.

Representative accessions were selected based on the advanced M strategy using a modified heuristic algorithm implemented in PowerCore software [30]. Categorical variables, such as genotype and qualitative phenotype were applied in several classes (3 to 12 classes) based on distinct characters. Continuous variables (quantitative phenotypes, 7 to 12 classes) were automatically classified into different categories in the software based on Sturges’ rule [31]. Therefore, a total of 264 phenotypic alleles were used to select the core entries (Additional file 1: Table S2).

### Evaluation of the core collections

To evaluate each core collection, diverse statistical indicators were calculated for two types of variables, continuous and categorical variables. For continuous variables, the percentage of significant difference between core collections and the entire germplasm collection was calculated based on the mean difference (MD) percentage, the coincidence rate (CR) of range, the variance difference (VD) percentage, and variable rate (VR) of coefficient of variation. Among the candidate core sets selected from each different data set, a core set with MD less than 20 % and CR more than 80 % was considered as a representative collection. In addition, a lower value in VD and higher value in VR was considered to indicate a more effective core collection [32]. For categorical variables, the I and J’ values were calculated and compared between the five core collections and the entire germplasm collection. The maximum value of I (I max) is calculated based on the log of the number of classes used in the entire collection; the value for a core collection should be comparable to that of the entire collection [8].

Three additional markers having multiple alleles, COS643, COS111, and L4RP-3 F, which were selected from the Sol Genomics Network [33] and Yang et al. [34], were used for validation of the core set. Melting curve patterns were identified by HRM analysis using a Rotor Gene 6000 (Qiagen, Valencia, CA, USA). Thermal cycling conditions were 10 min at 95 °C, 50 cycles of 3-step amplification profile of 20 s at 94 °C, 20 s at 55 °C, and 40 s at 72 °C, followed by final extension 60 s at 95 °C and 60 s at 40 °C. HRM analysis was performed increasing 0.1 °C for every two seconds from 70 to 90 °C.

Finally, the core collection (CC240) with the highest genetic diversity and evenness was planted in 2014 in a research farm (Suwon, Korea) to monitor the variation of the diverse traits. Morphological data were obtained for the same accessions that were genotyped. Thirty-two different traits related to plant habit (9), leaf (4), flower (6), fruit (10), and seed (3) were analyzed. Phenotype data were presented as the mean ± SE. The differences between the mean values of individual clusters were assessed using one-way ANOVA and Duncan’s multiple range tests. *P* < 0.05 was considered to indicate a statistically significant difference. The IBM SPSS Statistics v23 software (IBM Corp., Armonk, NY, USA) was used for analysis.

## Results

### Genetic diversity of the *Capsicum* germplasm

In our preliminary studies, a total of 4,652 non-redundant accessions from 11 species were screened using SNP markers to reveal the genetic diversity (Additional file 1: Table S3). Based on the H_O_ values, 673 accessions mostly from *C. annuum* with Ho value more than 0.3 were considered as F1 hybrids (Additional file 2) and excluded from analysis. In addition, 158 accessions with more than seven missing genotype data points were also excluded. Ultimately, a total of 3,821 accessions were used for further experiments (Table 1).

**Table 1:** Genetic diversity analysis of the 3,821 pepper accessions

Species	Number	H_O_	H_E_	I
*C. annuum*	3,383	0.12	0.44	0.69
*C. baccatum*	150	0.12	0.26	0.51
*C. cardenasii*	1	0.21	0.1	0.14
*C. chacoense*	24	0.17	0.28	0.54
*C. chinense*	105	0.11	0.38	0.56
*C. eximium*	3	0.14	0.23	0.45
*C. frutescens*	137	0.09	0.37	0.55
*C. galapagoense*	1	0.13	0.06	0.07
*C. praetermissum*	5	0.21	0.18	0.31
*C. pubescens*	11	0.16	0.12	0.29
*C. tovarii*	1	0.15	0.07	0.12
Total	3,821	0.15	0.23	0.38

*Ho* observed heterozygosity, *H*_*E*_ expected heterozygosity, *I* Shannon’s information index of diversity

Using the SNP genotyping results, the H_E_, H_O_, and I were calculated for 3,821 pepper accessions (Table 1). The H_E_ values ranged from 0.10 to 0.44, and I values ranged from 0.07 to a maximum of 0.69. The highest diversity values in *C. annuum* accessions (H_E_ = 0.44, I = 0.69) suggests that there is extensive genetic variation within this species. With the exceptions of *C. baccatum* and *C. pubescens*, the other domesticated species showed relatively high H_E_ values, above 0.37. The H_O_ value of *C. annuum* was 0.12, whereas those of the other species varied from 0.09 to 0.21. Four domesticated species *C. annuum*, *C. baccatum*, *C. chinense*, and *C. frutescens* and two wild species *C. chacoense*, and *C. eximium* had lower values for H_O_ compared to H_E,_ (Table 1) whereas *C. cardenasii*, *C. galapagoense, C. pratermissum*, *C. pubescens*, and *C. tovarii* had relatively higher values of H_O_ compared to H_E_. This pattern suggests that the first six species have experienced inbreeding for a long time which could be attributed to the interplay of many factors such as artificial selection, non-random mating between individuals, population structure and size, and Wahlund effect (mixing of individuals from different genetic sources) [35, 36]. By contrast, accessions of the latter five species were collected in different isolated locations where each accession had evolved independently.

### Population structure of the germplasm collection

**Fig. 1:**
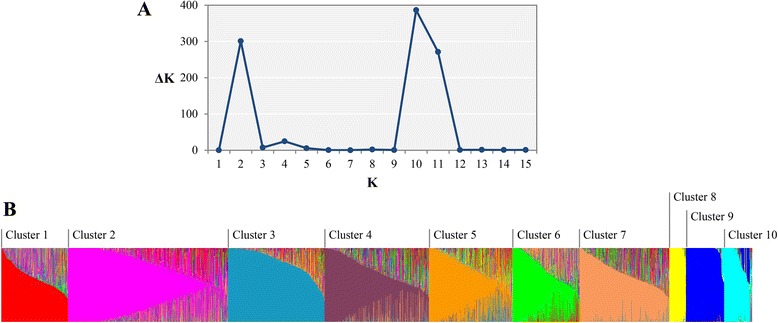
Population structure of the *Capsicum* germplasm collection. **a** ΔK reached its maximum value when K = 10 following the *ad-hoc* method. **b** Ten subpopulation clusters inferred by STRUCTURE are represented by different colors

The SNP genotyping results were used to perform population structure analysis for the 3,821 accessions under an admixed model using the STRUCTURE program [23]. Estimated likelihood (LnP (D)) was found to be greatest when K = 10, suggesting that the population used in this study can be divided into ten clusters (Fig. 1). The clusters 3, 8, 9, and 10 were rather well separated from others whereas the cluster 1, 2, 4, 5, 6, and 7 were admixtures. Each of the 10 clusters included different numbers of accessions, ranging from 85 to 806 (Table 2). The average distance (H_E_) between individuals in each cluster was 0.32. The highest H_E_ value of 0.43 was observed in cluster 5, indicating greater genetic diversity within this cluster, whereas cluster 9 showed the lowest H_E_ value of 0.11. Genetic differentiation (F_ST_) values varied from 0.08 to 0.78 with an average of 0.33. The smallest F_ST_ value (0.08) was observed in cluster 5, whereas cluster 9 had the highest F_ST_ value (0.78), indicating that accessions in this cluster have several different genotype patterns.

**Table 2:** Diversity-related summary statistics for all clusters inferred by STRUCTURE analysis

Cluster	Number	H_E_	I	F_ST_
1	341	0.41	0.61	0.13
2	806	0.34	0.53	0.27
3	487	0.37	0.57	0.22
4	535	0.40	0.60	0.16
5	426	0.43	0.66	0.08
6	341	0.35	0.54	0.27
7	461	0.33	0.51	0.31
8	85	0.20	0.34	0.57
9	196	0.11	0.19	0.78
10	143	0.24	0.40	0.49
Total	3,821	0.32	0.49	0.33

*H*_*E*_ expected heterozygosity, *I* Shannon’s information index of diversity, *F*_*ST*_ genetic differentiation

Most of the *C. annuum* accessions were found in clusters 1 to 7. *C. chinense* was mostly distributed in clusters 8 and 9, whereas *C. frutescens* was mostly found in clusters 4, 9, and 10. By contrast, *C. baccatum* was distributed in clusters 8, 9, and 10. *C. pubescens* was placed in cluster 10. Wild species *C. chacoense*, *C. cardenasii*, *C. eximium*, *C. praetermissum*, and *C. tovarii* were distributed in cluster 10 along with *C. baccatum* accessions (Additional file 1: Table S4). Although not fully distinct, the ten clusters were roughly separated according to geographic distribution. Clusters 1 to 3 were composed of an admixture of accessions from East Europe countries (Additional file 3). Cluster 4 to 7 were mostly composed of collections from East Asia. Interestingly, the Korean landraces belonged to clusters 6 and 7. The accessions of clusters 8 to 10 were mostly from South America.

### Molecular phylogenetic analysis of the germplasm collection

**Fig. 2:**
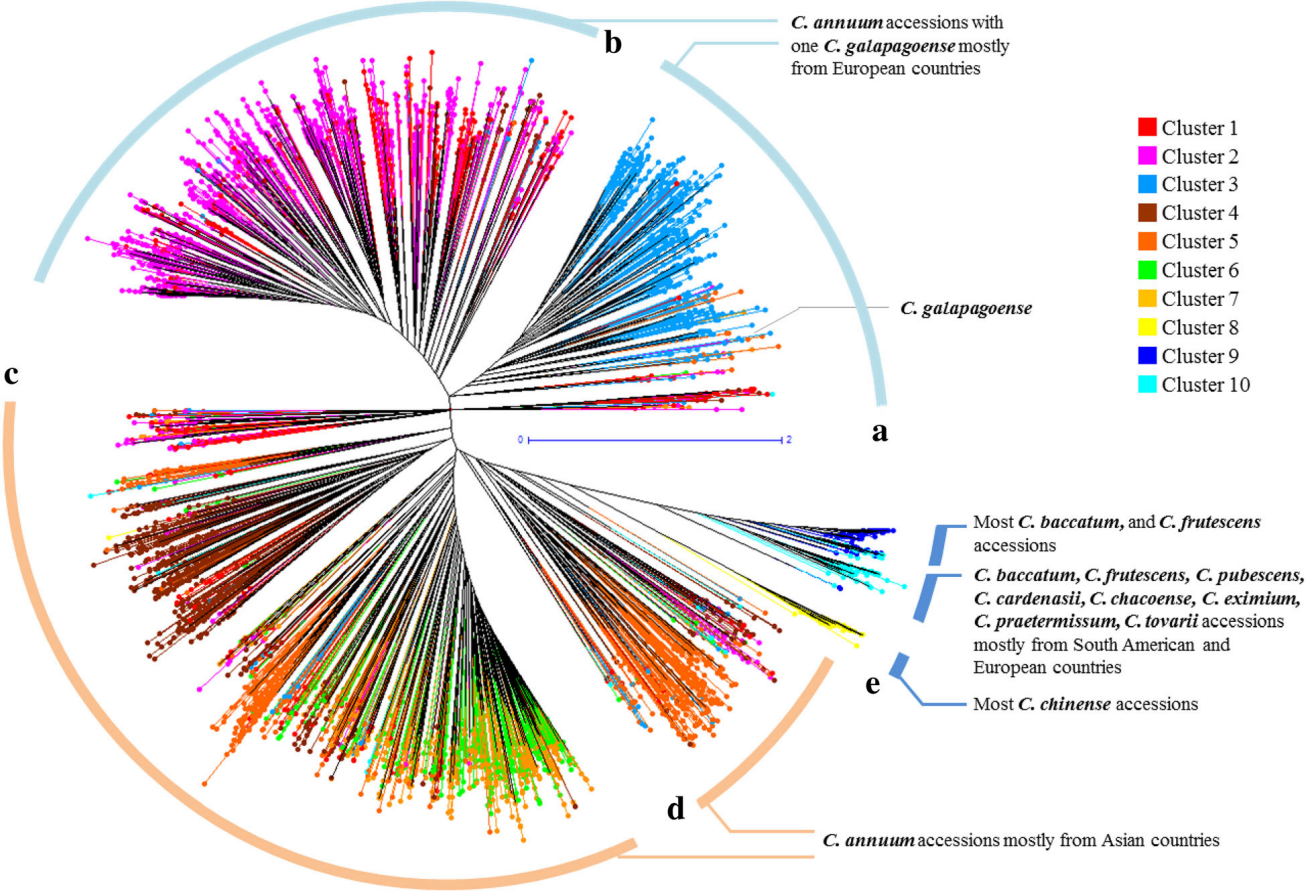
Unrooted phylogenetic tree of the *Capsicum* germplasm. The dendrogram was produced using the unweighted neighbor-joining method based on genetic dissimilarity among the 3,821 germplasm accessions. The colors of branches indicate accessions corresponding to the clusters (cluster 1 to 10) from population structure analysis as in Fig. 1. Five clades (**a**-**e**) were distinguished by distance between branches; **a** and **b** included *C. annuum* species mostly from Europe; **c** and **d** included *C. annuum* species mostly from Asia; **e** comprised other *Capsicum* species except *C. annuum*. **a** to **e** were used for a clustering range to select entries to construct the core collection

Using the genotyping data, an unrooted phylogenetic tree of the 3,821 pepper accessions was generated using the unweighted neighbor joining method based on genetic dissimilarity calculated with the Manhattan index. The tree showed five large clades (A-E) in which accessions of *C. annuum* were grouped separately from the other species (Fig. 2). The *C. annuum* accessions were found in four large clades. *C. annuum* accessions collected (or originated) in European countries were distributed among upper branches including the clades A and B. *C. annuum* accessions from Asian countries were distributed among lower branches (clades C and D). The accessions belonging to other species were clustered together in clade E. Within clade E, most of *C. chinense* accessions were clearly distinguished from those of other species and were placed next to clade D. When the unrooted phylogenetic tree was compared with the clusters obtained from the STRUCTURE analysis, the phylogenic tree matched well with the cluster separation in the STRUCTURE analysis. Accessions in cluster 3 belonged to clade A, accessions in clusters 1 and 2 to clade B, and accessions in clusters 4 to 7 to clade C; some of the accessions in cluster 5 and admixtures belonged to clade D and the accessions in clusters 8 to 10 were in clade E (Figs. 1 and 2). The unrooted phylogenetic tree more clearly differentiated groups according to their geographic origin.

### Optimization of core set selection methods and construction of the core collection

To determine the best possible method for selection of a core collection, five different sampling strategies were tested (Table 3). The first three methods were carried out using the entire collection without considering clustering analysis. These methods included selection of core entries based on SNP genotype data only (Gcc), phenotype data only (Pcc), or the combination of genotype and phenotype data (G + Pcc). In the other two methods, core entries were selected from each cluster of the unrooted phylogenetic tree. In these methods, after analysis of the entire collection, core entries in each cluster were selected based on genotype data only (Ggcc), or the combination of genotype and phenotype data (Gg + Pcc).

**Table 3:** Comparisons of distribution frequency and representativeness among five different core collections constructed based on diverse sampling strategies

Evaluated parameter		Data combination					Entire germplasm collection
		Gcc	Ggcc	Pcc	G + Pcc	Gg + Pcc	
Number of entries (%)		7 (0.2)	53 (1.4)	76 (2.0)	76 (2.0)	240 (6.3)	3,821
Number of alleles		96	96	264	360	360	360
Continuous variables	MD %	38.28	18.89	15.85	15.35	9.45	-
	CR %	13.95	50.89	96.99	97.04	98.40	-
	VD %	5757.70	80.02	49.37	46.55	32.46	-
	VR %	52.15	112.37	126.99	125.95	115.65	-
Categorical variables	I	0.88	0.91	0.94	0.97	0.95	0.85
	I max	1.25	1.25	1.25	1.25	1.25	1.25
	J’	0.77	0.79	0.78	0.81	0.80	0.73

Gcc: core collection constructed based on genotype with nongroup based strategy, Ggcc: core collection constructed from entries in each cluster grouped by genotype, Pcc: core collection constructed based on phenotype, G + Pcc: core collection constructed based on combination of genotype and phenotype, Gg + Pcc: core collection constructed based on genotype and phenotype combination from each cluster grouped by genotype. Distributional aspects to validate a representativeness of core collection; MD%: the mean difference percentage, CR%: the coincidence rate, VD%: the variance difference percentage, VR%: variable rate. Genetic diversity indices to validate categorical variables; I: Shannon’s information index of diversity, I max: logarithmic number of classes in entire collection, J’: genetics evenness

When only genotype data were used, 7 and 53 core entries were selected for Gcc and Ggcc, respectively. Ggcc showed a MD of less than 20 %, which is close to the mean value of the entire collection, whereas Gcc showed a MD of more than 20 %, poorly representing the entire collection. Both of them showed a CR of less than 80 %, demonstrating insufficient coverage of the phenotype variation of the entire collection. However, Ggcc exhibited a smaller percentage of VD and larger percentage of VR, which indicated that selection of a core set after clustering analysis (Ggcc) better represented the entire collection. Furthermore, the comparison of categorical variables including 48 SNPs markers and 15 qualitative traits produced a higher value in I for Ggcc (0.91) than for Gcc (0.88), but a similar value in J’ for Ggcc (0.79) and Gcc (0.77). Therefore, selecting the core entries after clustering analysis is more effective to represent the entire collection in terms of both phenotype and genotype data even using same number of alleles.

Since the core sets selected using only genotype data could not represent the diversity of the entire collection presumably due to limitations of number of SNP markers used, the available phenotype data for 32 traits were included for selection of core sets. Each trait included 3 to 12 phenotype classes providing at least 264 variations (Additional file 1: Table S2). A total of 76 entries were selected based on only phenotype data (Pcc) and produced 15.85 % in MD, 96.99 % in CR, 0.94 in I and 0.78 in J’, which reflects slightly better representation of the entire collection than that of Gcc. When both genotype and phenotype data were used (G + Pcc), the same number of entries (76), but slightly better representation of the entire collection was achieved compared to that with Pcc. As we found that selection of a core set after clustering analysis more effectively represented the entire collection, the final core collection was built using a combination of genotype and phenotype data after cluster analysis (Gg + Pcc). A total of 240 accessions representing six species, *C. annuum* (176), *C. baccatum* (21), *C. chinense* (22), *C. eximium* (2), *C. frutescens* (18), and *C. praetermissum* (1) were ultimately selected as a core collection (CC240) (Additional file 1: Table S5). Compared with the entire germplasm collection, CC240 showed 9.45 % in MD and 98.40 % in CR, which provided good coverage of most of the range of continuous phenotypes in the entire collection. Furthermore, CC240 showed the lowest MD and the highest CR of all tested core collections. In addition, the values of I and J’ were 0.95 and 0.80, respectively, which represents increased genetic diversity compared to the entire germplasm collection.

**Fig. 3:**
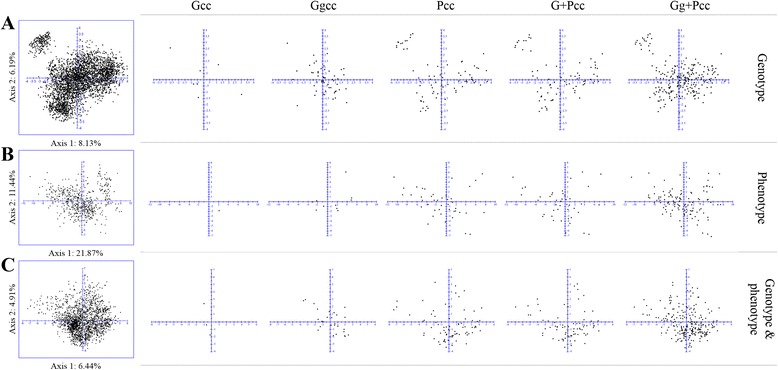
Principal coordinate analysis of distributions of diverse core entries selected from different sampling strategies. PCA was performed with DARwin 6.0.9 software based on distance matrices. **a** distribution of pepper accessions based on genotype included 3,821 accessions from entire germplasm collection, and 7, 53, 76, 76, and 240 accessions from Gcc, Ggcc, Pcc, G + Pcc, and Gg + Pcc sampling strategies, respectively; **b** distribution of pepper accessions based on phenotype included 589 accessions from entire germplasm collection, and 1,9, 42, 43, and 115 accessions from Gcc, Ggcc, Pcc, G + Pcc, and Gg + Pcc sampling strategies, respectively; **c** distribution of pepper accessions based on genotype and phenotype combination included 2,006 accessions from entire germplasm collection, and 3, 27, 76, 76, and 235 accessions from Gcc, Ggcc, Pcc, G + Pcc, and Gg + Pcc sampling strategies, respectively. Matrices surrounded with blue boxes indicate distributions of the pepper accessions from entire collection

To validate and confirm the distribution of core entries, core collections selected from five methods PCA was performed. The distribution of the entire germplasm collection and core collection entries on the basis of genotyping was explained by the first two principal components, where the first and second axes explained 8.13 and 6.19 % of the total variation among the accessions, respectively, and showed a clear separation of *C. annuum* from other species. PCA analysis based on phenotyping included 589 accessions with no more than 10 % of missing data points, whereas 2,006 accessions were plotted in the genotype plus phenotype background with no more than 20 % missing data to reach the least condition of unit pairing. In contrast to the phenotype (21.87 %, 11.44 %), the genotype (8.13 %, 6.19 %) and genotype plus phenotype (6.44 %, 4.91 %) revealed lower variation in each axis (Fig. 3). Overall, regardless of the selection method, PCA analysis showed that core entries were distributed evenly without obvious grouping, covering the variation of the entire germplasm collection.

### Evaluation of the core set using markers with multiple alleles

Evaluation of the quality of core collections should be based on data that were not used in the selection of the core set [37]. Accordingly, three additional multiple allelic markers, COS643, COS111, and L4RP-3 F, were used to evaluate the core set (Additional file 1: Table S6); the markers had 9, 16, and 9 alleles, respectively, in the entire germplasm collection (Table 4). The numbers of alleles in CC240 were the same as those in entire collection except for COS111 (11 instead of 16). The genetic diversity of CC240 revealed by these markers was compared with that of the entire collection. The average value for I in CC240 was higher (1.65) than that of the entire collection (1.50). Furthermore, the average genetic evenness was more stable in CC240 (0.74) compared to the entire collection (0.65). However, L4RP-3 F did not show a difference in genetic diversity or evenness because the I value of the entire collection (2.02) for that marker was already close to maximum value (I max = 2.20). In summary, the high genetic diversity and evenness of CC240 evaluated by three additional markers demonstrated that the core accessions in CC240 well represent the entire collection.

**Table 4:** Comparison of genetic diversity between the 3,821 accession collection and different core collections using an additional set of multiplex markers

Criteria	3,821 germplasm collection				CC240			
	COS643	COS111	L4RP-3 F	Avg.	COS643	COS111	L4RP-3 F	Avg.
Genotype patterns	9	16	9	-	9	11	9	-
I max	2.20	2.77	2.20	2.39	2.20	2.30	2.20	2.23
I	1.54	0.94	2.02	1.50	1.79	1.22	1.96	1.65
J’	0.70	0.34	0.92	0.65	0.81	0.53	0.89	0.74

I max: logarithmic number of classes in entire collection, I: Shannon’s information index of diversity, J’: genetic evenness

### Morphological variations of CC240

**Fig. 4:**
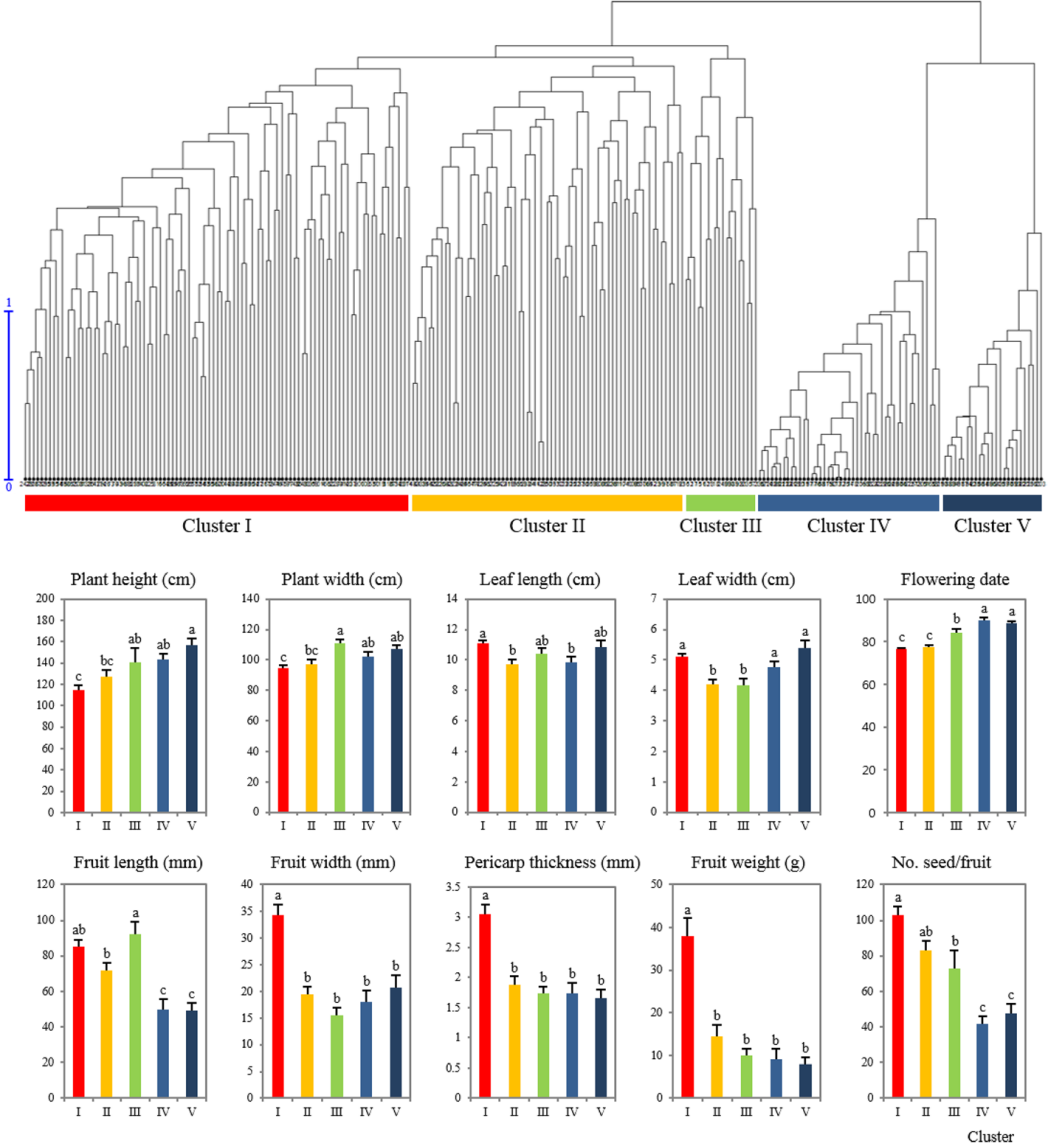
Comparison of phenotypic measurements among five clusters in CC240. Dendrogram was generated by hierarchical clustering (UPGMA) based on genetic dissimilarity. Average values of 10 different phenotypic characters (plant height, plant width, leaf length, leaf width, flowering date, fruit length, fruit width, fruit pericarp thickness, fruit weight, and number of seeds per fruit) were recorded to compare among the five clusters. Data are presented as the mean ± SE. *P* < 0.05 was considered to indicate a statistically significant difference, indicated by different lowercase letters

Accessions of CC240 were planted in an experimental farm and grown for 1 year to evaluate various traits. Four accessions, namely Javitott bogyiszloi (*C. annuum*), 9146 (*C. annuum*), Tabasco (*C. frutescens*), and 9148 (*C. frutescens*) were excluded from the phenotype analysis due to poor growth. Thus, phenotype evaluation was performed for 236 accessions for 32 different traits (Additional file 1: Table S2). Overall, CC240 showed a similar range of diversity in morphological traits as that of entire collection. For plant architecture, about one half of the accessions (105) showed the half-spreading phenotype. Plant height was varied between 40 cm to 200 cm, and plant width ranged between 25 cm to 130 cm. Leaf color varied from light green to dark green except for one accession having purple leaves; leaf length was 4.83 cm to 15.43 cm, and leaf width was 2.13 cm to 8.77 cm. Flower color of most accessions (188) was white, whereas 23 had light green flowers, and 21 had white flowers with yellow spots. Among all accessions, the earliest flowering date was 63 days from planting in *C. annuum* ‘Swedish’ and ‘A9E0211’, whereas the latest date was 103 days in *C. baccatum* ‘C01543’ and *C. annuum* ‘ACC160’. Length of fruit was distributed between 4.8 mm and 249 mm with an average of 72.66 mm, fruit width varied between 4.8 mm and 84.12 mm with an average of 24.82 mm, and thickness of pericarp was 0.2 mm to 7.2 mm. Fruit weight was distributed between 0.06 g and 177.32 g. There were five to 256 seeds in each fruit (Fig. 4).

Using genotype, accessions of CC240 were divided into five clusters (I-V) by hierarchical clustering (UPGMA) based on genetic dissimilarity of 48 SNP markers. Clusters I, II, and III included *C. annuum* species whereas clusters IV and V included other species, such as *C. baccatum*, *C. chinense*, *C. eximium*, *C. frutescens*, and *C. praetermissum*. Among the 32 morphological traits, 10 different quantitative traits including plant height, plant width, leaf length, leaf width, flowering date, fruit length, fruit width, fruit weight, number of seeds per fruit, and fruit pericarp thickness were significantly different between the clusters (Fig. 4). Cluster I was characterized by large-fruited peppers with thick pericarp. Leaves were large, plant height and width are slightly shorter, and flowering date was relatively earlier than that of *C. annuum* accessions in cluster III. Accessions in cluster II were characterized by small and short-fruited peppers. The pericarp of accessions in cluster II was thinner than that of those in cluster I, whereas plant height and width were slightly larger than those of cluster I. The flowering date of cluster II was similar to that of accessions of cluster I and much earlier than that of cluster III. Accessions in cluster III were characterized by elongated fruits. Plant height and width were larger and flowering date was later when compared with those of accessions in clusters I and II. The species included in clusters IV and V also exhibited differences in fruit shape, where slightly smaller fruit and thicker pericarp were observed for cluster IV. Plant height and width of cluster IV were smaller than those of accessions of cluster V. Slightly wider fruits with thinner pericarp were observed for cluster V. Leaf size was much larger, and the flowering date was slightly later than those of accessions of cluster IV. Overall, accessions in clusters IV and V exhibited small fruits with slightly higher plant height and late flowering.

## Discussion

Despite numerous *Capsicum* germplasm accessions having been documented worldwide, little is known about their population structure or genetic diversity in contrast to other crops. Previously, *Capsicum* germplasm collections have been examined for genetic diversity using accessions from Mesoamerica (Central Mexico to northwestern Costa Rica) to survey geographic origin and understand the domestication process [6, 38]. Recently, STRUCTURE analysis was performed in a *Capsicum* germplasm collection with 1,352 accessions, which was grouped into six distinct clusters based on genetic analysis with six SSR markers [14]. In the present study, a *Capsicum* germplasm collection consisting of 3,821 accessions was divided into ten clusters by STRUCTURE analysis and five distinct groups by phylogenetic analysis (Figs. 1 and 2). The AMOVA analysis revealed that the genetic variance among and within the populations was significant (*p* ≤ 0.01). Variance among populations and within a population of five phylogenetic groups were seven and 93 %, respectively and the variance among and within populations of ten STRUCTURE clusters were 31 and 69 %, respectively (Additional file 1: Table S7).

Both STRUCTURE and phylogenetic analyses showed that *C. annuum* accessions were separated from other species and sub-clustered into two large groups, one from European countries and the other from Asian countries. In comparison to the STRUCTURE analysis, the unrooted phylogenetic tree showed rather clear separation according to geographic origin and species classification. Accessions collected from Korea were spread in two clusters (clusters 6 and 7) as per the population structure analysis, whereas in the unrooted phylogenetic tree accessions corresponding to those two clusters were placed in a same node. Clade E included species other than *C. annuum* and showed distinct grouping. *C. chinense* accessions were separated out from other species and closely placed next to *C. annuum*. In pepper breeding, agriculturally useful traits such as disease resistance, fragrance, yield, and pungency have been introgressed from wild species by interspecific hybridizations. Among the domesticated species, *C. chinense* has better crossability with *C. annuum* and is used as a bridge species between *C. annuum* and other species [39, 40]. The location of *C. chinense* in this tree, between *C. annuum* and other species, may explain why *C. chinense* has played a role as an interspecific cross-bridge. Based the topology of the phylogenic tree, *C. annuum* accessions in clade D (Fig. 2) are candidate to be used as interspecific bridges to introgress genes from other species.

In this study, we confirmed that *C. galapagoense* was located more closely with *C. annuum* than other species. However, other species such as *C. baccatum*, *C. frutescens*, *C. pubescens*, and *C. chacoense* were not clearly separated from each other (Fig. 2). In previous work [14], classification with SSR markers showed rather clear distinction of species. It may indicate that SSR markers are more prone to be affected by speciation and evolution processes, whereas SNP markers are more appropriate for the analysis of genetic variation in various aspects of agronomic and morphological traits [41]. It is also possible that we did not use enough SNP markers to allow clear differentiation among species. In this study, we used 96 alleles to survey genetic diversity. The cost of genotyping of large germplasm collection is relatively expensive, therefore based on our preliminary studies with 412 SNPs [19], 48 SNPs with high PIC values were used for diversity study. Even though most of the SNP makers used in this study had high PIC values close to 0.5, SNP markers are less powerful than SSR markers in terms of relative kinship estimation and population structure analysis [42–44] because SSR markers have higher allelic diversity than SNP markers. To compensate for the small number of SNP markers, we also used 32 different traits which account for 264 phenotypic variations to build a core collection. Core collection built by more variations showed higher genetic diversity, evenness and representation. These results indicated that even with a small number of SNP markers used combination with diverse phenotypic data can be also effective to construct a core collection with the aim to conserve the phenotypic and genetic variability within species.

Representative core accessions have been selected in diverse crops using various sampling strategies combined with different clustering methods [15, 32, 45–47]. Among the strategies, the M strategy was reported to be a useful method in selecting a core set conserving high genetic diversity with a reasonable size [45]. There are two representative core selection methods implementing the M strategy, namely the MSTRAT algorithm [48] and PowerCore software [30]. Here, we used the advanced M strategy as implemented in PowerCore 1.0 software and successfully established a representative core collection with high genetic diversity. The advanced M strategy is based on the M strategy with heuristic searching that enables retention of all variations of the entire collection in the core collection with a minimum number of accessions. This strategy is more effective when using continuous variables in the dataset to capture a maximum of alleles with a minimum redundancy [30, 49].

Use of either genotype or phenotype information only for selection of core collection entries may not be efficient for capturing genetic diversity of the entire germplasm of a species. Therefore, we used both genotype and phenotype information along with clustering to select core collection entries. To determine the optimal core set selection methods, we compared five different methods and found that selection of the core set using genotype and phenotype data after clustering analysis (Gg + Pcc) is the best method (Table 3). Moreover, we investigated the relationship between the number of clusters and genetic diversity among different core sets in clustering analysis. Different combinations of clades A, B, C, D, and E from the unrooted phylogenetic tree (Fig. 2) and the 10 clusters from the population structure analysis (Fig. 1) were considered to select a core set. Core sets selected from the cluster combinations in tree were named CG3, CG4, and CG5, respectively and CST10 were from the 10 clusters in STRUCTURE analysis (Additional file 1: Table S8). From those core collections, 174 to 420 entries were selected. Every collection showed higher values of genetic diversity and evenness than the 3,821 germplasm collection; however, the core collections did not show statistically significant difference from each other (*P* > 0.05). Therefore, the number of clusters in the collection is not a critical factor to select highly diverse core entries.

To reveal the phenotype variation in CC240, core accessions were clustered into five distinct subclusters based on genotype relationship (Fig. 4). Among the five clusters, three of them (I, II, and III) represented *C. annuum* and other two (IV and V) included other species, such as *C. baccatum*, *C. chinense*, *C. eximium*, *C. frutescens*, and *C. praetermissum* without clear species distinction. In a previous study, *Capsicum* germplasm was divided into six clusters [14], in which three of them (1, 2, and 3) were composed of *C. annuum* accessions. Those three clusters were clearly distinguished mainly by fruit shape, such that cluster 1 was characterized by elongated fruited peppers, thin pericarp and late flowering, whereas cluster 2 exhibited conical fruit and rather thick pericarp, and cluster 3 had large-fruited peppers with thick pericarp and elongated-fruited peppers. Consistent with Nicolai’s work [14], three clusters in CC240 found in this study were characterized by large fruit with thick pericarp, large leaves, and early flowering date in cluster I, small, short fruit, small leaves, and early flowering date in cluster II, and elongated fruit with thin pericarp, late flowering date in cluster III. Thus, it appears that *C. annuum* accessions, which are mainly used as fundamental breeding materials, were clustered by breeding features based on food culture. Though, M strategy is the most powerful option for the selection of accessions with rich allelic diversity and for eliminating redundancies from noninformative alleles, it does not consider species composition while selecting core entries, which is a one of the disadvantage of the model based M strategy and therefore, future works should consider other measures of model fit including a rarefaction analysis, which corrects for sample sizes and manual inclusion of some representative wild species depending on the purpose of the core collection.

## Conclusions

Establishing a core collection of *Capsicum* will enhance the proper utilization of *Capsicum* genetic resources. In the present study, based on population structure, a core collection (CC240) of *Capsicum* was constructed using 48 SNP markers and 32 different traits. The core collection ‘CC240’ is composed of six *Capsicum* species from 44 geographic locations and was found to represent the diversity of the entire germplasm collection. This core collection will serve as a primary source for SNP mining and further genetic association and functional analyses for novel genes in *Capsicum*.

## Additional files

Additional file 1: Table S1. Summary of SNP markers used in the genetic analysis of pepper germplasm collection. **Table S2.** Description of various traits used in this study. **Table S3.** Genetic diversity analysis of the 4,652 pepper accessions. **Table S4.** Distribution of *Capsicum* species in STRUCTURE clusters within 3,821 germplasm accessions. **Table S5.** Accession in the ‘CC240’ core collection and group position of every accession in ‘CC240’ and the 3,821 germplasm collection. **Table S6.** Multiple allelic markers used to evaluate the core collection in *Capsicum.* **Table S7.** Analysis of molecular variance (AMOVA) among various subpopulations based on different clustering methods within 3,821 *Capsicum* accessions. **Table S8.** Comparison among different core collections established by diverse genetic clustering methods. CG3-CG5: Core collections based on unrooted phylogenetic tree clusters (A to E) grouped by 3 (A + B, C, D + E), 4 (A + B, C, D, E), and 5 (A, B, C, D, E) in respectively. CST10: Core collection based on 10 clustered population structure as in Fig. 1. Evaluated parameters; MD %: the mean difference percentage, CR %: the coincidence rate, VD %: the variance difference percentage, VR %: variable rate, I: Shannon’s information index of diversity, J': Genetics evenness, I max: logarithmic number of classes in entire collection. (XLSX 51 kb)
Additional file 2: Distribution of 4,652 *Capsicum* germplasm accessions based on H_O_ (observed heterozygosity). Accessions with an H_O_ value of more than 0.3 were considered as F1 hybrids. A total of 673 accessions were excluded from the fundamental germplasm collection to construct a core collection. (TIF 360 kb)
Additional file 3: Distribution of 3,821 germplasm accessions in population structure clusters according to their origin and geographic location. The colors of pie graph correspond to the clusters from STRUCTURE analysis as in Fig. 1. The area of each pie graph indicates the proportion of included accessions. (TIF 1168 kb)

## Abbreviations

CR: Coincidence rate of range
CTAB: Cetyl trimethylammonium bromide
F_ST_: Genetic differentiation
G + Pcc: Core entries based on the combination of genotype and phenotype data
Gcc: Core entries based on SNP genotype data only
Gg + Pcc: Core entries based on the combination of genotype and phenotype data
Ggcc: Core entries in each cluster were selected based on genotype data only
H_E_: Expected heterozygosity
H_O_: Observed heterozygosity
I: Shannon’s information index of diversity
J’: Genetic evenness
M strategy: Maximization strategy
MD: Mean difference percentage
Pcc: Core entries based on phenotype data only
PIC: Polymorphic information content
STA: Specific target amplification
VD: Variance difference percentage
VR: Variable rate of coefficient of variation
