# Supplementary figures and images for: Genetic diversity and population structure analysis to construct a core collection from a large Capsicum germplasm
Source: BMC Genet. 2016 Nov 14;17:142. doi: 10.1186/s12863-016-0452-8 (PMC5109817; doi:10.1186/s12863-016-0452-8)

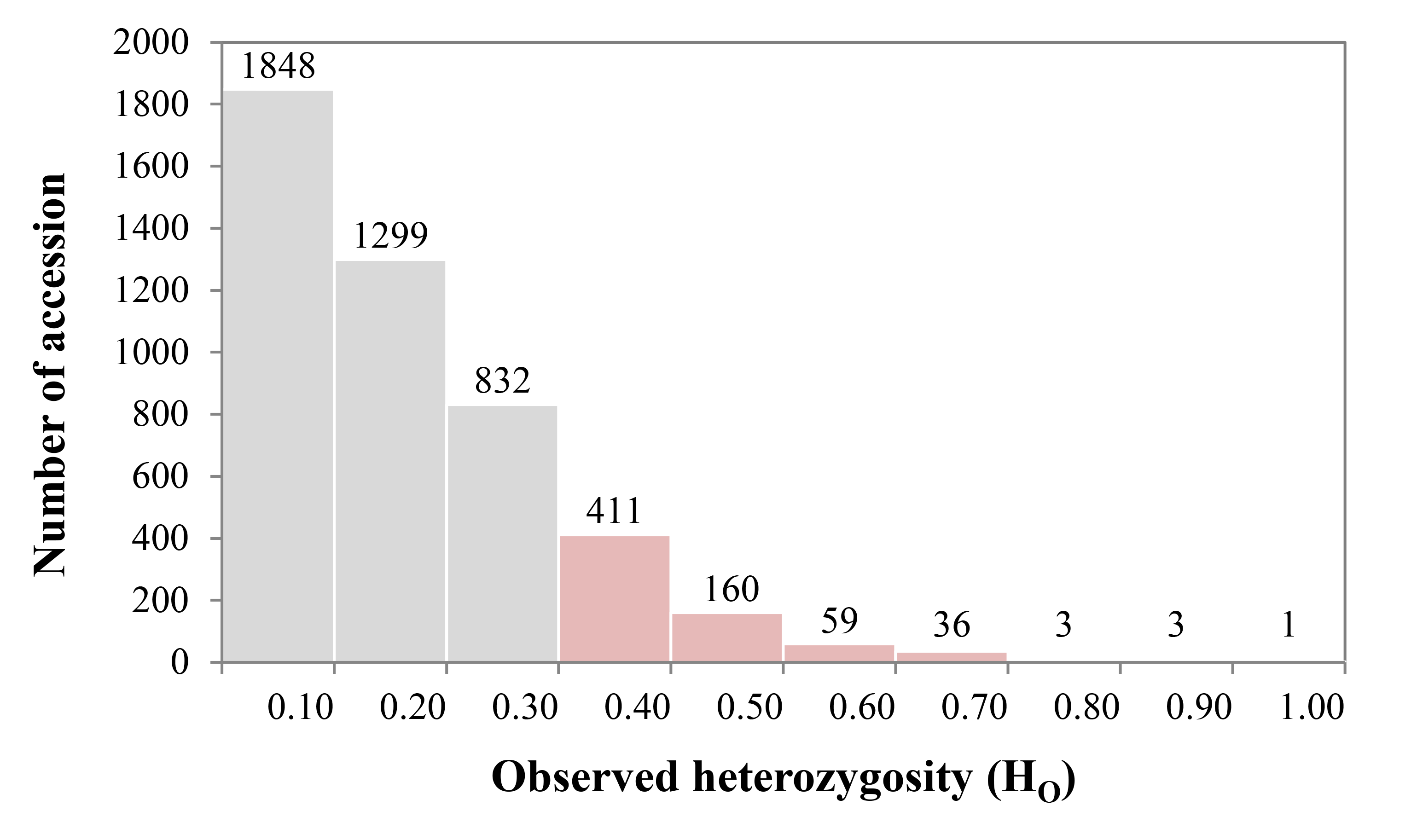

Supplement: Additional file 2: — Distribution of 4,652 Capsicum germplasm accessions based on HO (observed heterozygosity). Accessions with an HO value of more than 0.3 were considered as F1 hybrids. A total of 673 accessions were excluded from the fundamental germplasm collection to construct a core collection. (TIF 360 kb) [file 12863_2016_452_MOESM2_ESM.tif]

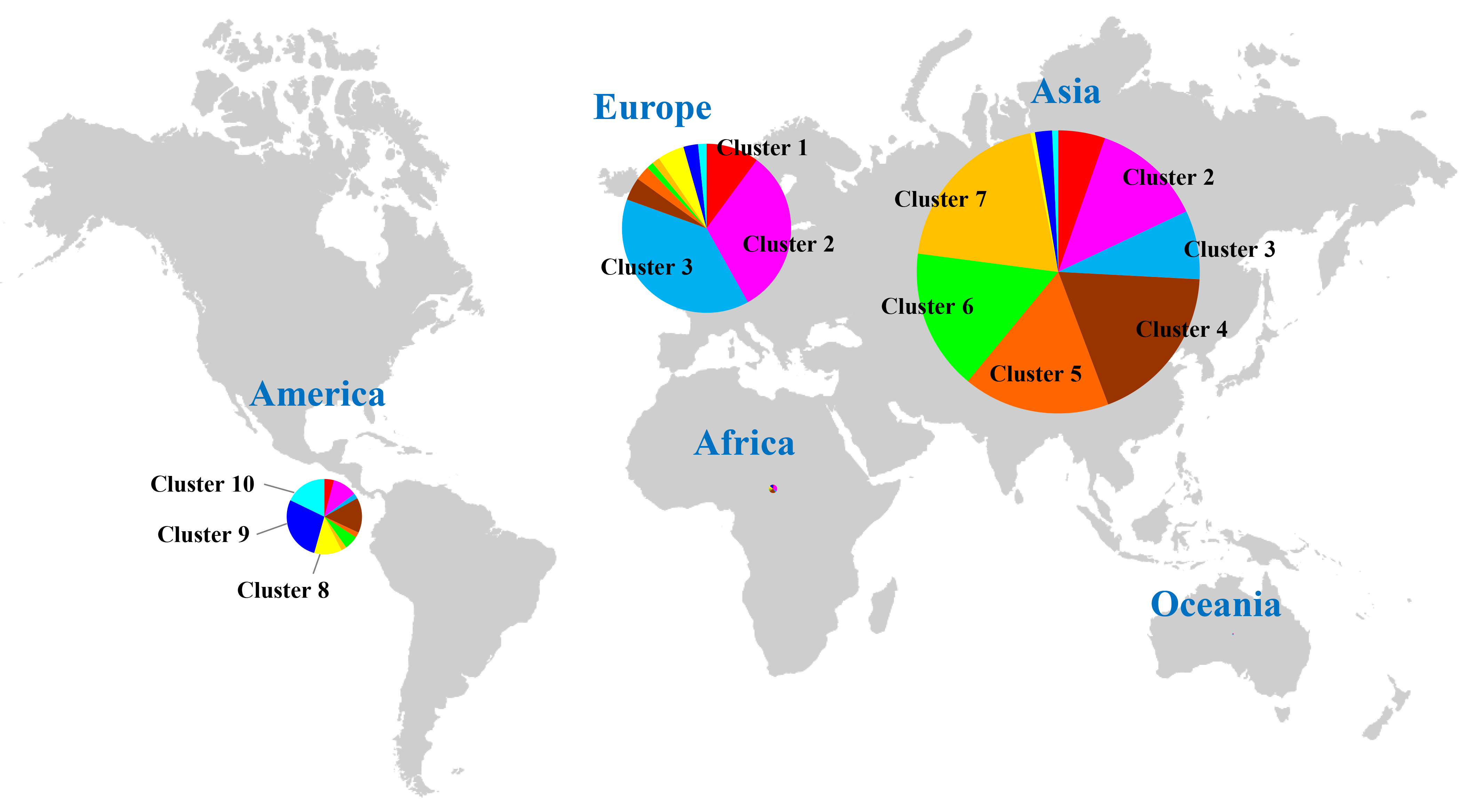

Supplement: Additional file 3: — Distribution of 3,821 germplasm accessions in population structure clusters according to their origin and geographic location. The colors of pie graph correspond to the clusters from STRUCTURE analysis as in Fig. 1. The area of each pie graph indicates the proportion of included accessions. (TIF 1168 kb) [file 12863_2016_452_MOESM3_ESM.tif]
